# Four Tendinous Slips of Flexor Hallucis Longus Tendon: A Case Report

**DOI:** 10.7759/cureus.59601

**Published:** 2024-05-03

**Authors:** Macie B Maddox, Sydney A Mashaw, Ellie M MacDonald, Andre J Mira, Weston J Parker, Adegbenro O Fakoya

**Affiliations:** 1 Anatomy, Louisiana State University Health Sciences Center, Shreveport, USA; 2 Cellular Biology and Anatomy, Louisiana State University Health Sciences Center, Shreveport, USA

**Keywords:** variant, plantar foot, tendon slips, flexor digitorum longus, flexor hallucis longus

## Abstract

Interindividual variability presents a rich field of study in medical sciences. During a cadaveric dissection at Louisiana State University Health Sciences Center, a rare anatomical variation was discovered in the pedal anatomy of a female cadaver. Medical students, while dissecting the sole of the foot, identified a variant tendinous structure. This aberrant tendinous slip from the flexor hallucis longus (FHL) extended to the lateral four tendons of flexor digitorum longus (FDL) along the plantar aspect of the foot. The discovery suggested that the FHL shares a functional relationship with the FDL. Application of tension to the FHL was found to result in simultaneous flexion motion in the lesser toes, from the second to the fifth digit. The presence of this anatomical variant holds considerable importance for surgical interventions, especially as a potential graft source in tendon reconstructions, warranting its documentation in this report.

## Introduction

The flexor hallucis longus (FHL) muscle originates from the posterior aspect of the fibula. The tendon of FHL continues through the tarsal tunnel, passing inferiorly to the medial malleolus of the tibia to insert at the distal phalanx of the great toe [1]. The FHL functions to flex the great toe and plantarflex the foot. In the tarsal tunnel, the FHL runs more posteriorly to the flexor digitorum longus (FDL), with the posterior artery, vein, and nerve in between. The FHL tendon, in its course, intersects with the FDL tendon at the master knot of Henry (MKH) [2]. The MKH is a point of anatomical crossover that was initially described by Arnold Kirkpatrick in 1945 and is now used interchangeably with chiasma plantare (CP) [3]. The MKH point is prone to “intersection syndrome,” including tendinosis, tenosynovitis, and tears [4].

Documentations of tendinous slip variations of the FHL around the chiasmatic point exist and are fairly common. However, the type of variation differs [5]. The variations have been categorized firstly by the type of connection between the FHL and the FDL. Type I is a proximal to distal connection from the FHL to the FDL. Type II is a proximal to distal connection from the FDL to the FHL. Type III is a crossed connection, and type IV is no connection between the FDL and FHL [5]. The variations are then further divided by the connections to the lesser toes with slips connecting to (a) the second toe, (b) the second and third toe, (c) the second through the fourth toe, and (d) all lesser toes [5]. The occurrence of these variations varied between studies based on the number of cadavers examined. In a study with 150 cadavers dissected, 28.7% were that of subtype (a), 52.0% were that of subtype (b), 18.7% were that of subtype (c), and 0.7% were that of subtype (d) [6]. Another study with 60 cadavers dissected showed that 33% were that of subtype (a), 55% were that of subtype (b), 7% were that of subtype (c), and 0% were that of subtype (d). In the same 60 cadavers, 67% were found to be type I, 3% type II, 30% type III, and 0% to be type IV [5].

The type of variation found within our cadaveric dissection is type 1d, where the tendon slip branches proximally from the FHL to the distal FDL with connection branches to digits two to five. This rare phenotype was found in only one cadaver in the aggregate cadaveric studies referenced above [5,6]. These slips have possible surgical implications as they could be used as donor tendons in lower extremity reconstruction, Achilles tendon rupture, posterior tibial tendon dysfunction, and fibularis longus/brevis rupture [3,4]. This case report shows a rare variation of the tendinous slip of the FHL into the FDL.

## Case presentation

**Figure 1:**
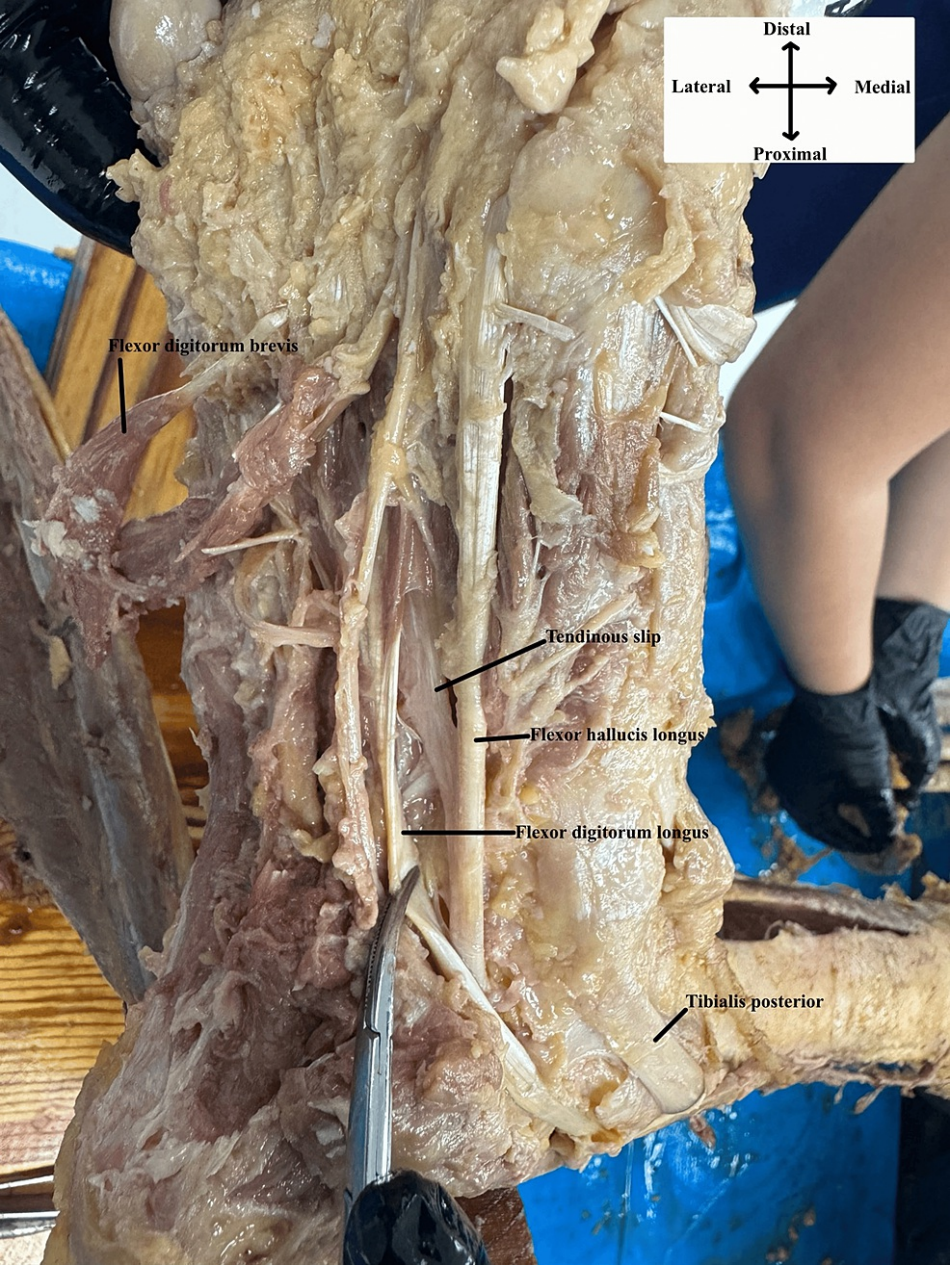
Origin of tendinous slip Plantar view of the 2nd and 3rd layer of the right foot showing the origin of the tendinous slip from the flexor hallucis longus tendon and its insertion into the flexor digitorum longus.

**Figure 2:**
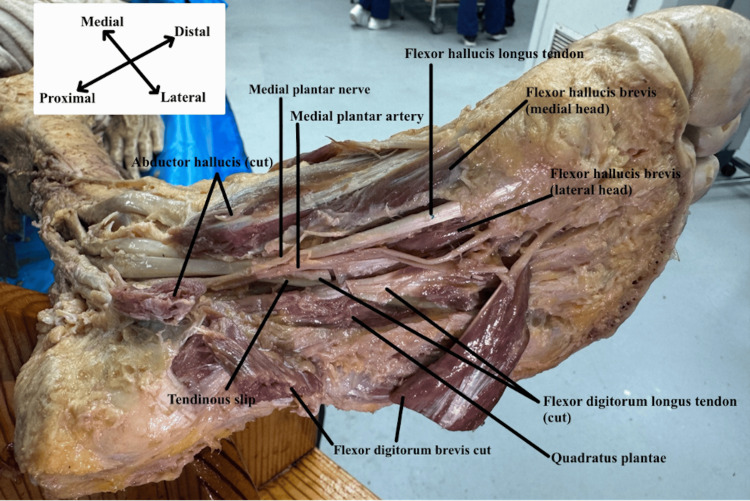
Tendinous slip with neurovasculature Plantar view of the left foot exposing the muscles and neurovasculature of the foot. The tendinous slip emerges deep into the medial plantar nerve and artery.

**Figure 3:**
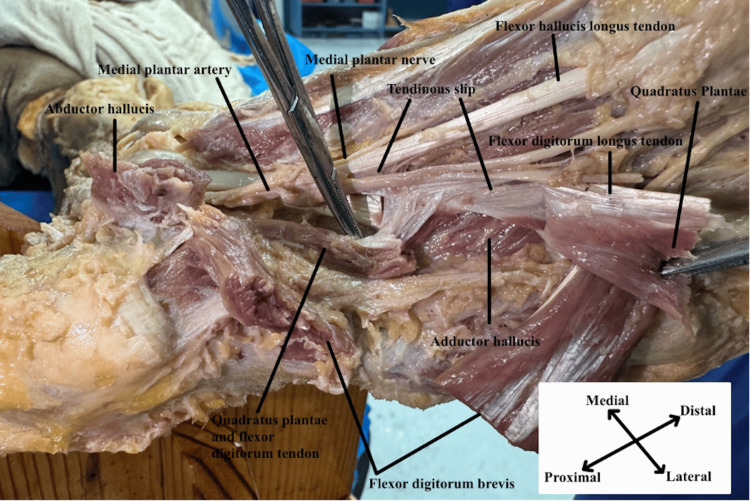
Full spectrum of the tendinous slip Plantar view of the left foot with flexor digitorum longus tendon and quadratus plantae muscle cut and reflected to show the 3rd layer of the sole of the foot. This layer also shows the full spectrum and insertion of the tendinous slip into the flexor digitorum longus tendon.

During proper dissection of the plantar surface of the feet in a 78-year-old white female cadaver, medical students at Louisiana State University Health Sciences Center, Shreveport School of Medicine, Louisiana, United States, discovered an anomalous variation involving the FHL tendon, FDL, and quadratus plantae. First, the skin, superficial fascia, and plantar aponeurosis were removed using sharp dissection to expose the first layer of the sole of the foot. Then, the muscle bellies of the flexor digitorum brevis (FDB) and abductor hallucis were transected proximally and reflected to expose the second layer. After this, a probe was placed underneath the FDL and quadratus plantae, and sharp dissection was used to carefully expose the third layer of the sole of the foot. Upon examination of the third layer, a unique variation was noticed regarding an extension of the FHL to the muscle belly of the quadratus plantae and the tendons of the FDL. There were extensions of the FHL for each tendon of the FDL, as shown sequentially in Figures 1-3. Additionally, when the FHL was pulled in tension, a flexure of all digits was seen. The remaining structures in the sole of the foot were normal. This rare variation was seen bilaterally.

## Discussion

The human body is a complex and intricate system, and the field of anatomy and cadaveric dissection continuously unveils new intricacies and connections. As previously stated, we found tendinous slips from the FHL to the FDL during a cadaveric dissection. In addition, we observed the movement of digits two to five when tension was applied to the base of the FHL tendon, suggesting that the FHL played a role in the movement of these additional digits. These intertendinous relationships have great clinical importance, especially in the field of foot and ankle surgery as well as rehabilitation.

Primarily, recognition of such anatomical variations is important to maintain precision when performing surgery on the foot, as well as decrease the likelihood of postoperative loss of function. Precise understanding is essential, as the tendon transfer involving the FHL and FDL is a common surgical intervention for conditions like Achilles tendinopathy and tendon ruptures [7]. Additionally, it finds applications in situations involving a tear in the fibularis tendons and flatfoot deformity due to an insufficient tibialis posterior tendon [8,5]. Tendinous slips between these tendons can help to maintain functional flexion in the digits following transfer [9]. Recent studies have even found that these common tendinous expansions can allow for longer, and thus more useful, tendon grafts for the treatment of such ailments [10]. The most common slip, a type 1 slip with a proximal-to-distal expansion of the FHL to FDL, provides the most beneficial use and is found in, on average, 85% of cadaveric dissections [11].

Another significant application of our discovery is regarding tendinous ruptures or lacerations. In cases where either the FHL or FDL tendons are damaged beyond functionality, the tendinous slips provide a level of redundancy that can help maintain functional flexion of the lateral four digits [12]. In the case of our cadaveric dissection, maintenance of some level of flexion could have been maintained in digits two to five. This is highly relevant in scenarios where preserving dexterity and mobility in the lateral toes is of utmost importance, such as in athletes or certain individuals with specific occupational demands [13]. These tendinous expansions act as a backup mechanism, allowing the intact tendon to compensate for the loss of function in the injured or lacerated one. Surgeons can then leverage this knowledge to devise surgical strategies that prioritize the preservation of these tendinous connections to optimize postoperative functional outcomes.

This discovery can also influence rehabilitation protocols following tendon repair. Physical therapists can tailor their interventions to promote the coordinated function of the intact tendons and their expansions, including targeted exercises to enhance the strength and coordination of the remaining tendons [14]. Over time, this can lead to full flexion of digits with tendinous slips, allowing patients to return to a level of normalcy and ensuring long-term functionality.

To summarize, our delve into the tendinous expansions from the FHL to the FDL through cadaveric dissection highlights the intricate web of anatomical connections made within the human body. These natural connections nearly always prove useful. Tendinous transfers prove useful in the treatment of many tendon malfunctions, and the redundancy of these slips helps to maintain function in the digits following such transfer. They also maintain functionality following tendon rupture or damage, which can be useful in patients requiring specific dexterity [15]. Surgeons can strategically use these interconnections to preserve function, while physical therapists can use rehabilitation practices to enhance flexion of the lateral digits. This connection improves surgical postoperative outcomes as well as long-term functionality in patients.

## Conclusions

In conclusion, although some anatomical variations are rare, they should be well documented. The authors herein present a unique case of tendinous expansions of the FHL tendon to the FDL tendon discovered in a female cadaver during dissection. This variant appeared to have some small overlapping functionality with the FDL tendon. Having a proper understanding of anatomical variants such as the one discussed in this case report may be used to improve patient results in various foot surgeries, such as Achilles tendon repair, tendon transfers, fibularis tendon repair, and surgical grafts. Along with this, understanding how unique anatomical variants impact the typical function of these muscles could be utilized to improve rehabilitation efforts and improve long-term patient outcomes.
